# Prolonged Survival of Allografts Induced by Mycobacterial Hsp70 Is Dependent on CD4+CD25+ Regulatory T Cells

**DOI:** 10.1371/journal.pone.0014264

**Published:** 2010-12-08

**Authors:** Thiago J. Borges, Bárbara N. Porto, César A. Teixeira, Marcelle Rodrigues, Felipe D. Machado, Ana Paula Ornaghi, Ana Paula D. de Souza, Fabio Maito, Wander R. Pavanelli, João S. Silva, Cristina Bonorino

**Affiliations:** 1 Faculdade de Biociências e Instituto de Pesquisas Biomédicas, Pontifícia Universidade Católica do Rio Grande do Sul, Porto Alegre, Brazil; 2 Departamento de Patologia Geral, Universidade Estadual de Londrina, Londrina, Brazil; 3 Departamento de Imunologia, Faculdade de Medicina de Ribeirão Preto, Universidade de São Paulo, Ribeirão Preto, Brazil; Fundação Oswaldo Cruz, Brazil

## Abstract

**Background:**

Heat shock proteins (Hsps) are stress induced proteins with immunomodulatory properties. The Hsp70 of *Mycobacterium tuberculosis* (TBHsp70) has been shown to have an anti-inflammatory role on rodent autoimmune arthritis models, and the protective effects were demonstrated to be dependent on interleukin-10 (IL-10). We have previously observed that TBHsp70 inhibited maturation of dendritic cells (DCs) and induced IL-10 production by these cells, as well as in synovial fluid cells.

**Methodology/Principal Findings:**

We investigated if TBHsp70 could inhibit allograft rejection in two murine allograft systems, a transplanted allogeneic melanoma and a regular skin allograft. In both systems, treatment with TBHsp70 significantly inhibited rejection of the graft, and correlated with regulatory T cells (Tregs) recruitment. This effect was not tumor mediated because injection of TBHsp70 in tumor-free mice induced an increase of Tregs in the draining lymph nodes as well as inhibition of proliferation of lymph node T cells and an increase in IL-10 production. Finally, TBHsp70 inhibited skin allograft acute rejection, and depletion of Tregs using a monoclonal antibody completely abolished this effect.

**Conclusions/Significance:**

We present the first evidence for an immunosuppressive role for this protein in a graft rejection system, using an innovative approach – immersion of the graft tissue in TBHsp70 solution instead of protein injection. Also, this is the first study that demonstrates dependence on Treg cells for the immunosuppressive role of TBHsp70. This finding is relevant for the elucidation of the immunomodulatory mechanism of TBHsp70. We propose that this protein can be used not only for chronic inflammatory diseases, but is also useful for organ transplantation management.

## Introduction

Heat shock proteins (Hsp) are highly immunogenic proteins, though conserved between mammals and microorganisms. Hsp70, originally described as a heat induced protein [1], is the most conserved of Hsps, and currently known to have immunomodulatory properties. Nevertheless, the exact mechanisms through which it exerts this effect are not completely clear [2]. *Mycobacterium tuberculosis* Hsp70 (TBHsp70) has been shown to protect from induced arthritis in rats [3], [4], [5]. We have demonstrated that TBHsp70 induces IL-10 production by monocytes and synovial cells of arthritis patients, leading to a reduction of TNF-α e IFN-γ levels [6]. Also, we observed that TBHsp70 can inhibit differentiation of bone marrow derived dendritic cells (BMDCs) *in vitro*, leading to the production of IL-10 by these cells [7]. It was suggested that exposure to bacterial Hsps could activate self Hsp-specific T cells that would be cross reactive with bacterial Hsps and trigger immunoregulatory pathways [8]. More recently, in a proteoglycan-induced arthritis model, TBHsp70 immunization showed a protective potential that was dependent on IL-10 [9]. In addition, the treatment with TBHsp70 upregulated IL-10 mRNA in regulatory T cells (Tregs).

Treg cells are crucial for the suppression of acute rejection in allografts [10]. These cells develop in the thymus or can be induced in peripheral sites when given appropriate signals by the antigen presenting cells. They are CD25+, and also express the transcriptional factor forkhead box 3 (FoxP3), cytotoxic T-lymphocyte antigen 4 (CTLA-4) and glucocorticoid-induced tumor necrosis factor receptor (GITR) [11]. They can suppress inflammatory responses by regulating the activity of self-reactive conventional T cells. Tregs produce IL-10 and TGF-β and actively suppress non-Treg proliferation [12]. They have been shown to be important for inhibition of allograft rejection in several models [13], [14]. In this study, we investigated whether TBHsp70 could act as an immunosuppressant in two allograft rejection systems. We also investigated if treatment with this protein would induce Treg cells. Our results suggest that TBHsp70 is capable of delaying acute allograft rejection, and this response is mediated by Tregs.

## Results

### TBHsp70 suppresses tumor allograft rejection

**Figure 1:**
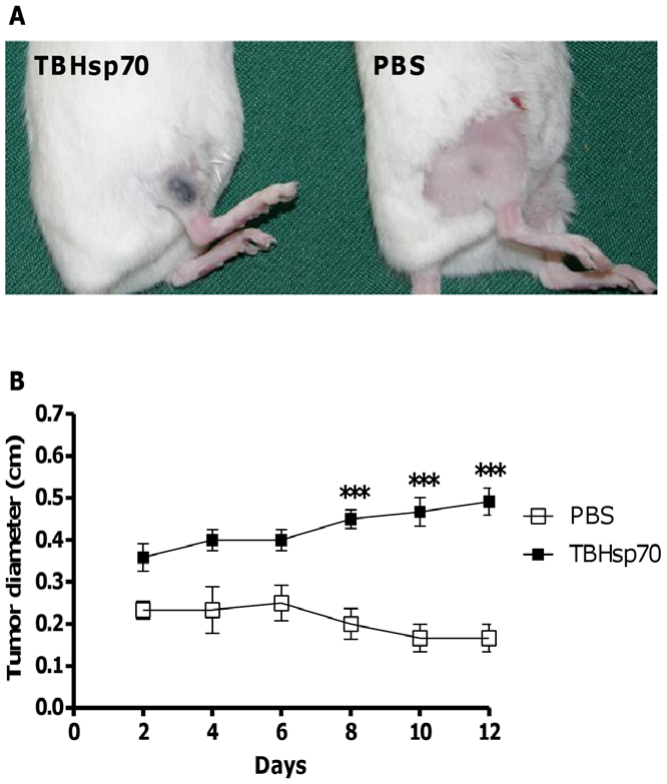
B16F10 tumor allograft in BALB/c mice. (A) Typical aspect of injection site in mice treated subcutaneously with tumor cells in PBS containing TBHsp70 or PBS alone on day 12 after transplant of tumor cells. (B) Sequential measurements of tumor diameter. n = 3 mice per treatment group. This experiment was performed seven times, with identical results. ***, p<0.0001.

We asked if TBHsp70 could inhibit allograft rejection in two different models. We first used a transplanted allogeneic tumor model, injecting BALB/c mice (H-2^d^) with B16F10 melanoma cells (H-2^b^). Cells were resuspended in PBS containing TBHsp70 or no stimulus and were injected subcutaneously in mice. Tumor growth was recorded daily for 12 days. Around day 6 after injection, PBS-injected mice completely rejected the tumor cells, and the progressive elimination of the dark area occupied by melanoma cells could be observed at the injection site (Fig. 1A). However, mice that received tumor cells with TBHsp70 not only did not reject the tumor (Fig. 1A), but also allowed tumor growth through the 12-day period (Fig. 1B). These results suggested that TBHsp70 could inhibit tumor allograft rejection, supporting the immunosuppressive potential that had been previously observed in arthritis models [3], [4], [15], [16].

### T cells with regulatory phenotype are observed at the tumor allograft site in HSP70-treated animals

**Figure 2:**
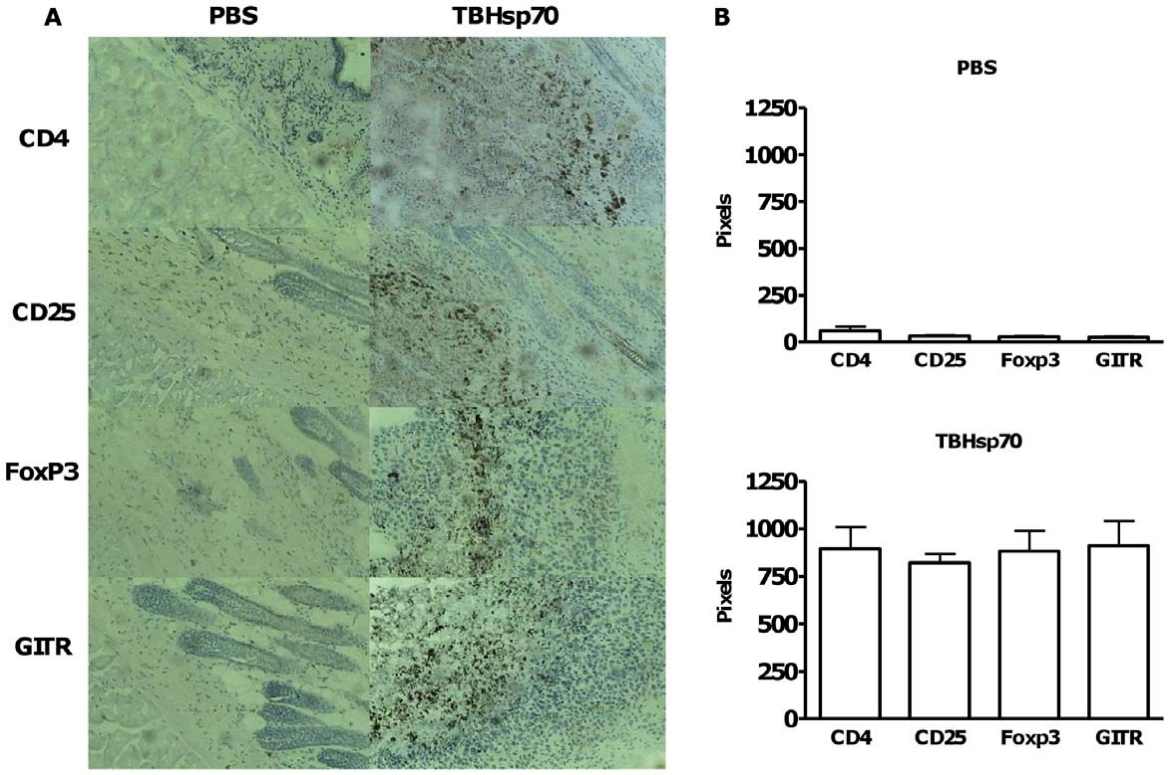
Treg cells are observed at the tumor allograft site. (A) Graft site (8 µm) serial sections from mice injected with tumor in PBS or TBHsp70 were stained with biotin-labeled anti-CD4, anti-CD25, anti-GITR and anti-Foxp3 antibodies, followed by streptavidin-peroxidase. Counter staining was hematoxillin. (B) Quantification of positive staining, expressed in pixels, using Image Pro Plus Software. n = 3 mice per group, experiments were performed 3 times.

We next investigated whether the inhibition of rejection of allogeneic tumor cells by TBHsp70 was associated with a local infiltration by Treg cells. We performed immunohistology on the tumor graft site, and sections were incubated with anti-CD4, anti-CD25, anti-FoxP3, and anti-GITR antibodies (Fig. 2). In mice injected with tumor cells in PBS alone, the tumor cells were not detectable by histology at the injection site, as analyzed by hematoxillin/eosin staining. Also, immunohistochemistry for CD4, CD25, FoxP3 and GITR was negative (Fig. 2A). In mice injected with tumor cells in TBHsp70 solution, staining for CD4, CD25, FoxP3 and GITR was observed in overlapping areas, mostly surrounding the tumor mass, as well as inside the tumor (Fig. 2A). This indicates that Tregs were locally infiltrating the tumor allograft site. Figure 2B shows the quantification of CD4, CD25, FoxP3 and GITR staining, captured with the Image Pro-Plus and quantified using the color range function in Adobe Photoshop tool.

### Injection of TBHsp70 suppresses T cell proliferation and induces Treg cells

**Figure 3:**
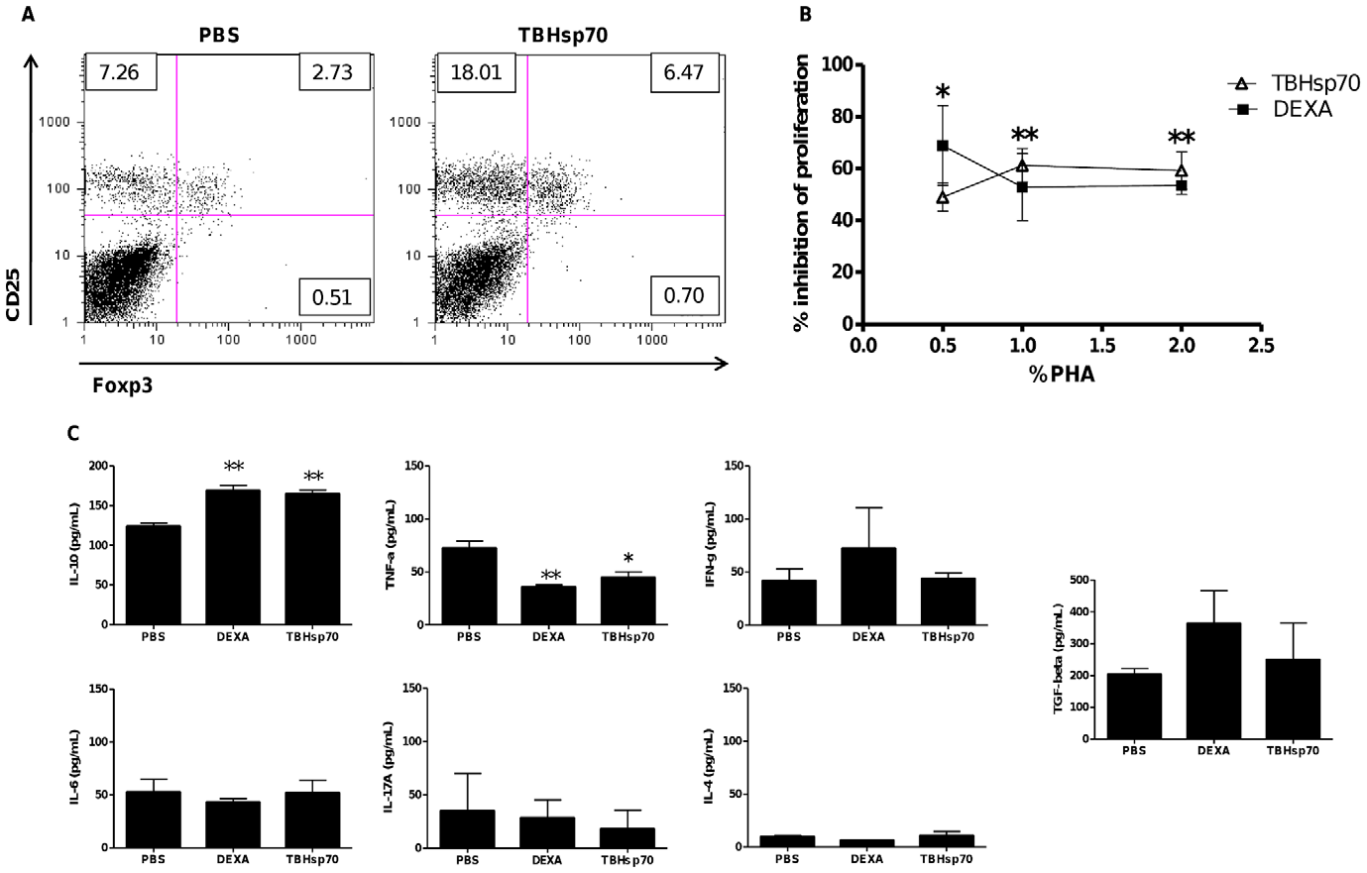
Local injection of TBHsp70 induces Tregs, IL-10 and leads to suppression in the draining lymph nodes. (A) Mice were injected subcutaneously in the thigh with PBS or 30 µg of TBHsp70. Draining lymph nodes were excised 4 days later, and cells were stained with fluorescent antibodies for CD4 and CD25, or CD4 and Foxp3. Plots show events gated on CD4+ cells. (B) Mice were injected subcutaneously in the thigh with TBHsp70 (1.5 mg/kg), DEXA (0.25 mg/kg) or PBS. Draining lymph nodes were excised 4 days later and single cell suspensions of lymph nodes were stimulated *in vitro* for 4 days with 0, 0.5, 1, or 2% of PHA. Viability of cells proliferating in response to PHA was estimated by an MTT assay, O.D. being read at 570 nm. Data are expressed as percentage of PHA-inhibited proliferation considering the O.D. at each PHA concentration as 100% proliferation. (C) Cytokine production of cultures supernatants was analyzed by flow cytometry, using a CBA (Mouse Th1/Th2/Th17) kit, except for TGF-β production, which was analyzed by ELISA. *, p<0.05; **, p<0.01.

Tumors can develop varied immunosuppressive strategies in order to grow [17], and those include the recruitment of Tregs [18], [19], [20]. It was possible that the Tregs observed infiltrating the TBHsp70 treated tumors could be a result of tumor activity, rather than a direct effect of TBHsp70. To investigate this possibility, tumor-free mice were injected subcutaneously with TBHsp70 (30 µg) or PBS, and after 4 days they were sacrificed. Draining lymph nodes were excised and a single cell suspension was obtained. Cells were counted and stained for the presence of Treg cells, with anti-CD4 Cychrome, anti-Foxp3 PE and anti-CD25 FITC, and analyzed by flow citometry. The injection of TBHsp70 led to a 2.4-fold increase in the percentage of CD25+ Foxp3+ cells among CD4+ lymphocytes compared to the PBS-injected animals (Fig. 3A). Because the total number of cells was also increased two-fold in the lymph nodes of tumor-treated animals, the total increase in CD4+ CD25+ Foxp3+ was approximately four-fold, compared to the PBS-injected animals (Fig. 3A). This result supported our hypothesis that TBHsp70 could induce Treg cells in the draining lymph node.

The lymph nodes are the sites to which antigens from the periphery are drained by the lymphatic system [21], and subcutaneous injection of antigens results on the presentation of such antigens to T cells in the draining lymph nodes [22]. Cell interactions occurring in the draining lymph nodes are thus crucial to the immune responses leading to allograft rejection or acceptance [23]. To determine whether the immunosuppressive effects of TBHsp70 would affect T cell proliferation in the draining lymph node, we injected mice subcutaneously with PBS, TBHsp70 or Dexamethasone (DEXA) as a positive control for suppression of proliferation. Four days later, the draining inguinal lymph nodes were excised, a single cell suspension was obtained and the cells were cultured with PHA for another four days. Viability of proliferating cells was estimated by an MTT assay. As expected, mice injected with DEXA exhibited significant inhibition of polyclonal T cell proliferation (Fig. 3B). Surprisingly, TBHsp70 inhibition of lymph node cell proliferation was superior to the inhibition induced by DEXA (Fig. 3B). Supernatants from cell cultures were analyzed for the presence of IL-10, TNF-α, IL-4, IL-6, IFN-γ, IL-17A and TGF-β. IL-10 production was significantly upregulated by treatment with TBHsp70 as well as by the treatment with DEXA, and both treatments significantly inhibited TNF-α production (Fig. 3C). No differences were observed for the other cytokines analyzed, including TGF-β. These results indicated that TBHsp70 treatment leads to suppression of lymph node T cell proliferation *in vitro*, and this is associated with the upregulation of IL-10 production and inhibition of TNF-α.

Altogether, the results suggested that TBHsp70 could induce Tregs which correlated with the induction of IL-10 in the draining lymph node, and that was independent of the presence of tumor cells.

### TBHsp70 treatment delays skin allograft rejection

**Figure 4:**
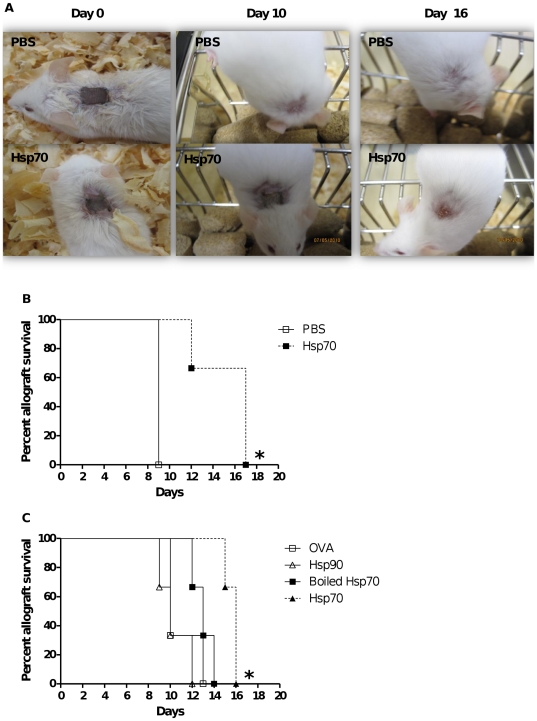
TBHsp70 delays skin graft rejection. (A) Skin grafts were immersed in a PBS solution (500 µl) containing TBHsp70 (30 µg) or PBS alone for 60 minutes at 4°C. After this, the skin graft was sutured to the exposed tissue of the recipient. Animals were kept in individual mini-isolators and observed daily, the state of graft acceptance being photographed and recorded. Graft rejection was confirmed by the observation of cyanosis, erythema, erosion, and loss of skin graft. (B) Survival curve of skin allograft immersed in PBS alone or TBHsp70 (30 µg). (C) Skin grafts were immersed in a PBS solution (500 µl) containing Ovalbumin (30 µg), Hsp90 (30 µg), 30 µg of TBHsp70 boiled for 10 minutes at 100°C or native TBHsp70. *, p<0.05. n = 3 mice per treatment group, the experiments were performed 4 times.

To further characterize the immunosuppressive effect of TBHsp70 over allograft rejection, and to eliminate the tumor cell variable, we switched to a traditional skin allograft model. We transferred a section of tail skin from C57BL/6 mice to BALB/c mice. The donor skin was immersed in a PBS solution containing TBHsp70 (30 µg in 500 µl, or 60 µg/ml) or PBS alone for 60 minutes at 4°C, and the graft site was analyzed daily. These experimental conditions were chosen because we had previously tested the injection of TBHsp70 at the graft site, either before or after performing the graft, and immersion of the donor graft in the TBHsp70 solution showed the best effect for a single TBHsp70 treatment (data not shown). We also tested immersing the graft in 20, 40 and 60 µg/ml of TBHsp70 solution, and the latter concentration presented the best results. At day 9, mice that received the skin grafts treated with PBS alone completely rejected the graft (Fig. 4A, B). On the other hand, mice receiving TBHsp70-treated skin graft presented prolonged acceptance of graft until day 17 (Fig. 4B). These results suggested that treatment with TBHsp70 significantly delays skin allograft rejection (*p = 0.0455*). To confirm the specificity of the regulatory effects observed with TBHsp70 treatment, we repeated the graft experiments using different proteins. Hsp90 was used to demonstrate that the effect is not a general property of Hsps, but rather of TBHsp70. Ovalbumin is a largely used antigen control, and was also used by us to treat the skin before grafting. Finally, we treated skin fragments with TBHsp70 boiled for 10 min at 100°C, to exclude the possibility that the effect could be due to a heat-resistant contaminant (such as LPS has been described to be [24], [25]). Once again, only TBHsp70 treatment was capable to prolong the allograft acceptance (Fig. 4C, *p = 0.0246*).

### Treg cells are essential for skin allograft survival induced by TBHsp70

**Figure 5:**
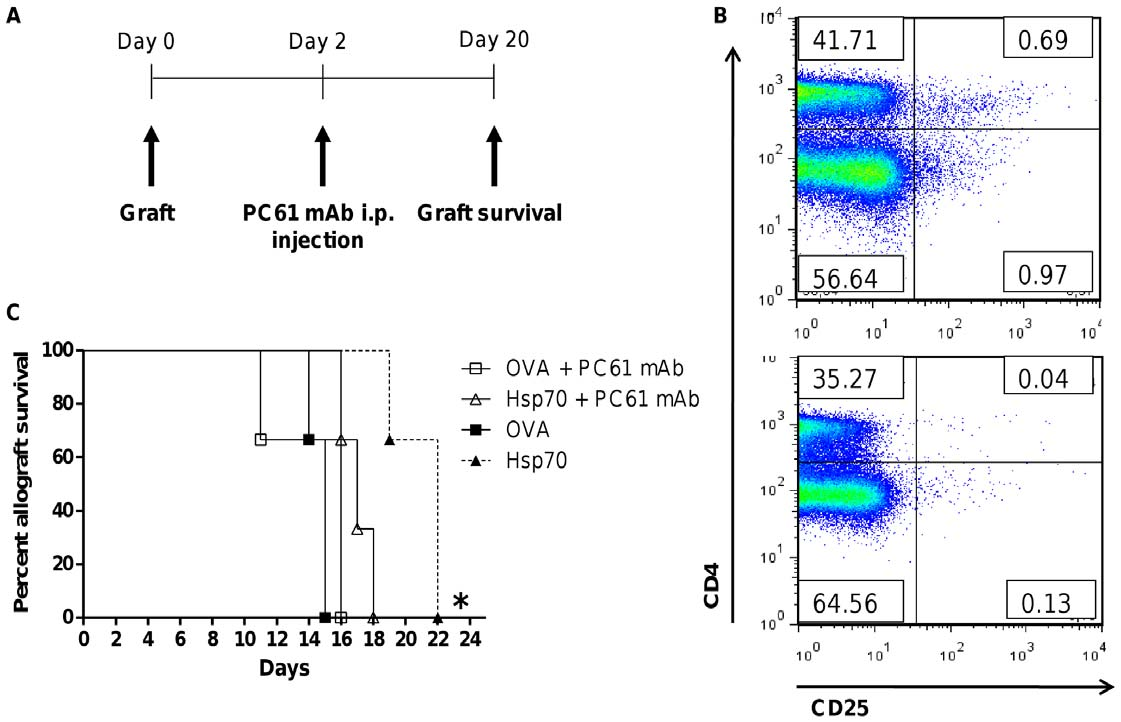
CD4+CD25+ regulatory T cells are crucial for prolonged survival induced by TBHsp70. Mice were injected i.p. with a single injection of 150 µg anti-CD25 mAb (PC61). (A) Schematic representation of *in vivo* Treg depletion and transplantation. (B) For depletion confirmation, lymph nodes were excised, collagenase D treated, stained with anti-CD4 and anti-CD25 and analyzed by flow cytometry. (C) Survival curve of skin allograft immersed in PBS containing 30 µg of OVA or TBHsp70. Groups were CD25+ depleted or not depleted. *, p<0.05. n = 3 mice per treatment group. Depletions were performed 2 times.

We next asked whether TBHsp70-prolonged survival of skin allograft was mediated by Tregs. To investigate this possibility, we depleted these cells by treating recipient BALB/c mice with anti-CD25 mAb (PC61). Depletion with one injection of this antibody is known to eliminate ∼70% of Tregs [26]. We performed a single mAb injection two days post-transplant (Fig. 5A). This treatment resulted in ∼95% depletion of CD4+CD25+ T cells (Fig. 5B).

Depletion of CD25+ T cells in TBHsp70 treated mice completely abolished the suppressive effect (Figure 5C). Once again, TBHsp70-treated skin allografts had a significant prolonged survival compared to OVA-treated grafts (*p = 0.0295*). Interestingly, depletion of Treg cells diminished this effect. Mice that were treated with PC61 mAb and TBHsp70 showed decreased graft survival compared to the TBHsp70-treated, not depleted mice (*p = 0.0246*). Taken together, these results indicate that the TBHsp70-induced delay of skin graft rejection is mediated by Treg cells.

## Discussion

In this study, we investigated the ability of TBHsp70 to inhibit allograft rejection in two different models as well as the mechanism mediating this effect. Although TBHsp70 exerts suppressive effects in inflammatory diseases, the mechanism by which it does so has not been fully elucidated. However, the induction of IL-10 production and the suppression of inflammatory cytokines seem to be consistent findings associated with TBHsp70 immunomodulatory effects [5], [9], [15].

The immunoregulatory role performed by Tregs is also frequently associated with the production of IL-10 [27], [28]. We demonstrated here, for the first time, that the immunosuppressive effect of TBHsp70 depends on Tregs. This was correlated with production of IL-10 in the draining lymph nodes. We have demonstrated in previous studies that TBHsp70 can induce IL-10 production by monocytes [6] and immature DCs [7]. Thus, it is possible that the IL-10 detected in our experiments is not solely produced by Tregs. This needs to be ascertained in further experiments with intracellular staining. Because Tregs can be induced locally by DCs with an immature phenotype that produce IL-10 [13], [29], [30], it is possible that TBHsp70 modulates the phenotype of DCs, inducing a regulatory response, and IL-10 production by DCs, leading to the recruitment and/or induction of Tregs. The importance of donor DC modulation for graft acceptance and Treg generation has been demonstrated in different systems [30], [31], [32].

Importantly, we verified that the induction of IL-10 in lymph nodes of TBHsp70 injected mice correlated with inhibition of TNF-α. This result corroborates an observation made by us in a previous study, in which in vitro treatment of synovial cells from arthritis patients with TBHsp70 inhibited TNF-α and induced IL-10 production [6]. This ability is likely to be determinant for the immunosuppressive effects verified for this protein both in arthritis and in the graft system. Interestingly, TGF-β, another cytokine commonly linked to Treg activity (or specific subsets of Tregs – see review in [33]) does not seem to be involved in this process, at least not in our experimental system.

We cannot conclude from the evidence presented here that the Tregs observed are specific for TBHsp70. It has, however, been hypothesized, that Hsp70 peptides constitute ligands for Treg cells [8]. This hypothesis is not completely excludent from the first one. Nevertheless, we believe that additional studies are necessary to discern from these two hypotheses, both in arthritis and transplant systems, to verify if the effect can be reproduced exclusively with TBHsp70 peptides. Because TBHsp70 immunosuppressive effects occur in rats as well as in mice, with different MHC elements, it could be predicted that multiple peptides of this protein would be candidate ligands. However, it is also possible that such peptide ligands do exist, and yet it is still necessary to initiate the response with DC modulation by TBHsp70 in its native structure. Indeed, the abrogation of suppressive effect of TBHsp70 by boiling suggests this might be a possibility. If so, the immunomodulatory effect could probably be reproduced with adoptive transfers of DCs treated with TBHsp70, and antigen processing inhibition in this system could indicate the relevance of TBHsp70 peptide presentation for the induction of Tregs. We are currently performing such experiments.

An attractive and innovative feature of TBHsp70 treatment in our system was that it was local, rather than systemic. All immunosuppressive drugs are delivered systemically, leading to undesirable side effects [34], [35], [36]. We verified that local treatment with TBHsp70 had a local effect, though it still remains to be determined if any systemic alterations were induced. Also, we were able to observe a significant difference with only one dose of the protein, while most immunosuppressants are used daily. Further studies need to be performed to determine how long additional doses of the protein will extend survival and acceptance of the graft.

Finally, we observed that the best effect on prolonged skin allograft survival was obtained immersing the graft in a TBHsp70 solution. The solutions used for preservation of organs before transplant are mainly buffered saline solutions that aim to protect from ischemia/reperfusion damage [37], [38], [39]. Although the endogenous Hsp70 protects organs against ischemia [40], [41], we do not believe that this was the mechanism of action involved in the inhibition of rejection in our study, because it was abolished by depletion of Tregs. Nonetheless, our results suggest that this protein could be used in the preservation solution of solid organs, conferring an additional benefit in the use of these preparations, delaying acute rejection.

## Materials and Methods

### Mice

Female BALB/c and C57BL/6 mice between 6–8 weeks old were purchased from FEPPS (Rio Grande do Sul, Brazil). All animals were housed in individual and standard mini-isolators (Techniplast, Italy) in an SPF facility (Faculdade de Biociencias – PUCRS) and had free access to water and food. All procedures were previously reviewed and approved by the Ethics Committee for the Use of Animals of Pontifícia Universidade Católica do Rio Grande do Sul (CEUA-PUCRS) under protocol ID CEUA 08/00048.

### Protein purification and LPS extraction

Recombinant TBHsp70 was produced in XL1-blue *Escherichia coli* (*E.coli*) (a gift from Dr Douglas Young, Hammersmith Hospital, London, UK), and purified according to Mehlert [42]. Estimation of protein concentration and its purity was performed against a BSA standard curve on a 10% SDS-PAGE gel stained with Coomassie blue. To remove LPS, Triton X-114 was used according to the method described in Aida [43]. Briefly, 5 µl of Triton X-114 (Sigma) were added to 500 µl recombinant protein. After vortexing vigorously, the solution was incubated in ice for 5 min, vortexed again and incubated at 37°C for 5 min. The solution was then centrifuged for 5 minutes at 37°C and the supernatant collected and the procedure repeated 5 more times. Contaminating Triton was removed by incubating overnight with Biobeads (Bio-Rad) at 4°C with agitation. To test for remaining contaminant LPS, a bioassay was performed. BALB/c mice were injected i.v. with 100 µl volume of either PBS alone, PBS with 40 µg of LPS (Sigma) or PBS with 40 µg TBHsp70. Mice were sacrificed 6 hours after injection. The spleens were removed, collagenase D treated, and the single cell suspensions obtained were analyzed for CD11c and CD86 expression by flow cytometry, as described in Khoruts et al. [44]. Preparations were considered LPS free only when CD86 was not upregulated in splenic DCs.

### Cell proliferation/viability assay

T cell proliferative responses were determined by a modified colorimetric assay [45]. To analyze inhibition of murine cell proliferation in vitro, mice were injected subcutaneously in the thigh with TBHsp70 (1.5 mg/kg), DEXA (0.25 mg/kg) or PBS. Draining lymph nodes were excised 4 days later and single cell suspensions of lymph nodes (8×10^5^/ml) were cultured with 0, 0.5, 1, or 2% of PHA for 4 days.

For the MTT assay, in the last 4 h of culture, 100 µl of the supernatant was gently discarded and 30 µl of freshly prepared MTT (3-(4,5-diamethyl 2-thiazolyl) 2,5 diphenyl-2H-tetrazolium - Sigma) solution (5 mg/ml in RPMI 1640 - Sigma) was added to each well. The cell cultures were incubated for 4 h at 37°C in 5% CO_2_ atmosphere. After completely removal of the supernatant, 100 µl of DMSO (Sigma) was added to each well. The optical density (OD) was determined using a Biorad ELISA plate reader at a wavelength of 570 and 620 nm. The viability of proliferating cells was expressed as the percentage of inhibited PHA-induced proliferation.

### Cytokine measurement

To analyze the profile of cytokine production, mice were injected subcutaneously in the thigh with TBHsp70 (1.5 mg/kg), DEXA (0.25 mg/kg) or PBS. Draining lymph nodes were excised 4 days later and single cell suspensions of lymph nodes (8×10^5^/ml) were cultured with 5 µg of conA for 4 days.

Murine cell culture supernatants were analyzed by a CBA mouse Th1/Th2/Th17 kit by flow cytometry using a FACSCalibur (Beckton Dickinson) according to manufacturer's instructions. TGF-β measurements were made using a Human/Mouse TGF-B1 ELISA Ready-Set-Go! Kit (eBioscience).

### Tumor injections and measurements

B16F10 cells were cultured in complete DMEM (Sigma) with 10% fetal calf serum (FCS). BALB/c mice were anesthetized (intraperitoneally) with a 100 µl volume of PBS 34% ketamine, 10% xylazine and fur from the upper thigh was removed. Cultured tumor cells that were 80% confluent were detached from the tissue culture plate with PBS 15 mM EDTA, washed, counted and ressuspended to a density of 1.5×10^6^ cells in 150 µl PBS containing either LPS-free TBHsp70 (30 µg) or PBS alone. These mixtures were injected subcutaneously in the outer region of the thigh of mice. Tumor diameter was measured with a caliper and photographed daily, for a period of two weeks. These experiments were repeated six times.

### Immunohistology

Lymph nodes and thighs of the tumor-injected animals were embedded in tissue-freezing medium (Tissue-Tek - Miles Laboratories), and stored in liquid nitrogen. Serial cryostat sections were mounted on poly-L-lysine-covered glass slides and fixed for 10 min in cold acetone, washed in PBS, and incubated for 30 min in a wet chamber at room temperature with PBS and normal goat serum (Sigma-Aldrich) diluted 1/50 to reduce nonspecific binding and then incubated for 40 min with biotinylated anti-CD4, anti-CD25, anti-GITR or anti-FoxP3, according to a previous titration assay. Next, sections were incubated with avidin-biotin-peroxidase complex, the color developed with 3,3′-diaminobenzidine (Vector Laboratories). The slides were counterstained with Mayer hematoxylin, dehydrated, and mounted with Canada Balsam. The software used to capture and count the cells was the Image Pro-plus version 4.1.5 (Mediacybernetics), which uses a video camera connected to a computer card to capture the images of the selected microscopic field. Structures were selected on the computer screen with the mouse pointer and then counted manually. Histological sections were captured by ZEISS – Axioskope 40 microscope equipped with a CoolSNAP-PRO color camera. Positive cells to CD4, CD25, GITR and FoxP3 were counted at 400x magnification, and photographs had the number of pixels quantified in each section, using the Image Pro Plus Software (version 4.1.5, Media Cybernetics Inc., Bethesda) and the color range Adobe Photoshop tool. Histology was performed 3 times, in different experiments.

### Skin Graft Model

For the experimental model of skin graft, the proceeding described by Billingham and Medawar was employed [46]. Briefly, C57BL/6 donor mice were sacrificed, 1 cm^2^ sections of tail skin were removed and immersed in a PBS solution (500 µl) containing TBHsp70 (30 µg), or PBS alone for 60 minutes at 4°C. Control treatments were PBS solutions (500 µl) containing either 30 µg of Hsp90 (StressGen), 30 µg of Ovalbumin (Sigma) or 30 µg of TBHsp70 boiled for 10 minutes at 100°C. BALB/c recipient mice were anesthetized as described above, and fur was shaved off the dorsal trunk. At the shaved area, 1 cm^2^ of skin was removed in each recipient mouse. One donor tail skin fragment was sutured to the exposed tissue of each recipient. Animals were kept in individual cages and observed daily, the state of graft acceptance being photographed and recorded. Graft rejection was confirmed by the observation of cyanosis, erythema, erosion, and loss of skin graft. Each experiment was performed four times.

### *In vivo* Tregs depletion

Mice were injected i.p. a single injection containing 150 µg of anti-CD25 mAb, purified from PC61 hybridoma culture supernatant using a protein A column (Sigma), or with PBS. The injection was given 2 days after skin transplantation. PC61 hybridoma cells were kindly provided by Dr. Ross Kedl, National Jewish, Denver, Colorado. Efficiency of depletion was analyzed by flow cytometry of lymphoid organs, staining for CD4, CD25 and FoxP3.

### Flow cytometry

Lymph nodes from mice injected with different treatments were excised after animals were sacrificed, and the organs disrupted against a nylon screen in media containing Collagenase D (Roche). Single cell suspensions were obtained, cells counted with Trypan blue and stained with antibodies against CD4-cychrome, CD25-PE and Foxp3-biotin, followed by streptavidin-FITC; or CD11c-FITC and CD86-PE (all purchased from Pharmingen). Cells were analyzed in a FACSCalibur flow cytometer (Bekton Dickinson).

### Statistical analysis

Statistical analysis was performed using the Prism software (version 5.00, Graphpad Software Inc., San Diego). The one-way ANOVA test was used to determine differences between groups. Multiple comparisons among levels were checked with Bonferroni post hoc tests. Differences between specific points were determined by a t test. To analyze skin graft survival, the Kaplan-Meier method was used. The level of significance was set at *p<0.05*.
